# Increasing genetic representation globally to advance Alzheimer's disease solutions for all

**DOI:** 10.1002/alz70855_106725

**Published:** 2025-12-24

**Authors:** Kyle M Scott, Farid Rajabli, Joshua O Akinyemi, Larry D Adams, Motunrayo Coker, Kazeem Akinwande, Samuel Diala, Patrice G Whitehead, Mayowa Ogunronbi, Kara L Hamilton‐Nelson, Albertino Damasceno, Andrew F Zaman, Yared Z Zewde, Biniyam A Ayele, Allison M Caban‐Holt, David Ndetei, Anthony J Griswold, Fred Stephen Sarfo, Susan H. Blanton, Albert Akpalu, Michael L Cuccaro, Kolawole Wahab, Katalina F. McInerney, Reginald Obiako, Olusegun Baiyewu, Pedro R Mena, Njideka U Okubadejo, Mayowa O Owolabi, Jenny Chavez, Izri M Martinez, Brian W Kunkle, Jeffery M Vance, Christiane Reitz, Giuseppe Tosto, Scott M Williams, William S Bush, Raj Kalaria, Jonathan L Haines, Adesola Ogunniyi, Goldie S Byrd, Rufus O Akinyemi, Margaret Pericak‐Vance, African Dementia Consortium (AfDC), Alzheimer's Disease Sequencing Project (ADSP)

**Affiliations:** ^1^ University of Miami Miller School of Medicine, Miami, FL, USA; ^2^ Dr. John T. Macdonald Foundation Department of Human Genetics, University of Miami Miller School of Medicine, Miami, FL, USA; ^3^ John P. Hussman Institute for Human Genomics, University of Miami Miller School of Medicine, Miami, FL, USA; ^4^ College of Medicine, University of Ibadan, Ibadan, Oyo, Nigeria; ^5^ Cell Biology and Genetics Unit, Department of Zoology, University of Ibadan, Ibadan, Nigeria; ^6^ Faculty of Medicine, Eduardo Mondlane University, Maputo, Mozambique; ^7^ College of Health Sciences, Addis Ababa University, Addis Ababa, Ethiopia; ^8^ Wake Forest University School of Medicine, Winston‐Salem, NC, USA; ^9^ Africa Institute of Mental and Brain Health, Nairobi, Kenya; ^10^ Komfo Anokye Teaching Hospital, Kumasi, Ghana; ^11^ University of Ghana Medical School, Accra, Ghana; ^12^ Department of Medicine, University of Ilorin, Ilorin, Nigeria; ^13^ Department of Neurology, University of Miami Miller School of Medicine, Miami, FL, USA; ^14^ Neurology Unit, Department of Medicine, Ahmadu Bello University/Ahmadu Bello University Teaching Hospital, Zaria, Nigeria; ^15^ Institute for Advanced Medical Research and Training, College of Medicine, University of Ibadan, Ibadan, Nigeria; ^16^ College of Medicine, University of Lagos, Lagos, Nigeria; ^17^ Institute for Advanced Medical Research and Training, College of Medicine, University of Ibadan, Ibadan, Oyo, Nigeria; ^18^ Gertrude H. Sergievsky Center, Taub Institute for Research on the Aging Brain, Departments of Neurology, Psychiatry, and Epidemiology, College of Physicians and Surgeons, Columbia University, New York, NY, USA; ^19^ Case Western Reserve University School of Medicine, Cleveland, OH, USA; ^20^ Cleveland Institute for Computational Biology, Department of Population & Quantitative Health Sciences, School of Medicine, Case Western Reserve University, Cleveland, OH, USA; ^21^ Translational and Clinical Research Institute, Newcastle University, Newcastle, United Kingdom

## Abstract

**Background:**

The “Recruitment and Retention for Alzheimer's Disease Diversity Genetic Cohorts in the Alzheimer's Disease Sequencing Project” (READD‐ADSP) is building a resource aimed at increasing genetic ancestry representation in Alzheimer's disease (AD) studies to advance our ability to leverage all genetic variation in solving AD for everyone. READD‐ADSP includes four US sites and ten countries in sub‐Saharan Africa part of the Africa Dementia Consortium (AfDC). The main goal is to characterize AD genetic risk and protective factors to understand AD mechanisms that can inform prevention and treatment globally. In this resource, we explored known AD loci using genetic data from West and East Africa: Nigeria, Ghana, Kenya, Ethiopia and Mozambique.

**Method:**

We performed genome‐wide association analysis on 275 AD cases and 278 cognitively unimpaired controls. We estimated population substructure using PC‐AiR, which is robust to known and cryptic relatedness. We investigated known AD loci from non‐Hispanic White (NHW) and African American (AA) genome‐wide association studies (Bellenguez et al. and Ray et al.). For association analysis, we employed a mixed‐model regression approach (SAIGE) where we controlled for age, gender, population substructure, and relatedness. To further explore genetic diversity, we calculated global ancestry using ADMIXTURE.

**Result:**

Admixture and principal component analysis revealed distinct ancestral patterns between West and East Africa, as well as substantial differential distributions of variation within each region. Among the previously reported lead genetic markers in NHW and AA GWAS studies, only *APOE e4* (OR=2.0; CI:1.4‐3.0; *p* = 3.1x10^‐8^) reached genome‐wide significance. Three additional loci (*ABCA7, BCKDK/KAT8, TREML2*) showed nominal significance for AD risk.

**Conclusion:**

Our findings confirm the role of *APOE e4* in AD risk among African ancestries and its smaller effect size relative to NHW populations, and replicate three additional loci (*ABCA7, BCKDK/KAT8, TREML2*) that show nominal associations with AD. Admixture and principal component analysis underscores the extensive genetic variability among the African diaspora. Ongoing data collection through the AfDC and READD‐ADSP at large to reach 13,000 total participants across global populations will continue to enhance understanding of AD pathogenesis. By leveraging genetic diversity from multiple populations, we can ultimately accelerate the discovery of interventions that benefit all communities.

